# Estimated transmissibility and impact of SARS-CoV-2 lineage B.1.1.7 in England

**DOI:** 10.1126/science.abg3055

**Published:** 2021-03-03

**Authors:** Nicholas G. Davies, Sam Abbott, Rosanna C. Barnard, Christopher I. Jarvis, Adam J. Kucharski, James D. Munday, Carl A. B. Pearson, Timothy W. Russell, Damien C. Tully, Alex D. Washburne, Tom Wenseleers, Amy Gimma, William Waites, Kerry L. M. Wong, Kevin van Zandvoort, Justin D. Silverman, CMMID COVID-19 Working Group, COVID-19 Genomics UK (COG-UK) Consortium, Karla Diaz-Ordaz, Ruth Keogh, Rosalind M. Eggo, Sebastian Funk, Mark Jit, Katherine E. Atkins, W. John Edmunds

**Affiliations:** 1 Centre for Mathematical Modelling of Infectious Diseases, London School of Hygiene and Tropical Medicine, London, UK.; 2 Selva Analytics LLC, Bozeman, MT, USA.; 3 Lab of Socioecology and Social Evolution, KU Leuven, Leuven, Belgium.; 4 College of Information Science and Technology, Pennsylvania State University, University Park, PA, USA.; 5 Centre for Statistical Methodology and Department of Medical Statistics, London School of Hygiene and Tropical Medicine, London, UK.; 6 Centre for Global Health, Usher Institute of Population Health Sciences and Informatics, University of Edinburgh, Edinburgh, UK.

## Abstract

A severe acute respiratory syndrome coronavirus 2 (SARS-CoV-2) variant, VOC 202012/01 (lineage B.1.1.7), emerged in southeast England in September 2020 and is rapidly spreading toward fixation. Using a variety of statistical and dynamic modeling approaches, we estimate that this variant has a 43 to 90% (range of 95% credible intervals, 38 to 130%) higher reproduction number than preexisting variants. A fitted two-strain dynamic transmission model shows that VOC 202012/01 will lead to large resurgences of COVID-19 cases. Without stringent control measures, including limited closure of educational institutions and a greatly accelerated vaccine rollout, COVID-19 hospitalizations and deaths across England in the first 6 months of 2021 were projected to exceed those in 2020. VOC 202012/01 has spread globally and exhibits a similar transmission increase (59 to 74%) in Denmark, Switzerland, and the United States.

## UK variant transmission

Severe acute respiratory syndrome coronavirus 2 (SARS-CoV-2) has the capacity to generate variants with major genomic changes. The UK variant B.1.1.7 (also known as VOC 202012/01) has many mutations that alter virus attachment and entry into human cells. Using a variety of statistical and dynamic modeling approaches, Davies *et al.* characterized the spread of the B.1.1.7 variant in the United Kingdom. The authors found that the variant is 43 to 90% more transmissible than the predecessor lineage but saw no clear evidence for a change in disease severity, although enhanced transmission will lead to higher incidence and more hospital admissions. Large resurgences of the virus are likely to occur after the easing of control measures, and it may be necessary to greatly accelerate vaccine roll-out to control the epidemic.

*Science*, this issue p. eabg3055

## Abstract (structured)

**INTRODUCTION:**

Several novel variants of severe acute respiratory syndrome coronavirus 2 (SARS-CoV-2), the virus that causes COVID-19, emerged in late 2020. One of these, Variant of Concern (VOC) 202012/01 (lineage B.1.1.7), was first detected in southeast England in September 2020 and spread to become the dominant lineage in the United Kingdom in just a few months. B.1.1.7 has since spread to at least 114 countries worldwide.

**RATIONALE:**

The rapid spread of VOC 202012/01 suggests that it transmits more efficiently from person to person than preexisting variants of SARS-CoV-2. This could lead to global surges in COVID-19 hospitalizations and deaths, so there is an urgent need to estimate how much more quickly VOC 202012/01 spreads, whether it is associated with greater or lesser severity of disease, and what control measures might be effective in mitigating its impact. We used social contact and mobility data, as well as demographic indicators linked to SARS-CoV-2 community testing data in England, to assess whether the spread of the new variant may be an artifact of higher baseline transmission rates in certain geographical areas or among specific demographic subpopulations. We then used a series of complementary statistical analyses and mathematical models to estimate the transmissibility of VOC 202012/01 across multiple datasets from the UK, Denmark, Switzerland, and the United States. Finally, we extended a mathematical model that has been extensively used to forecast COVID-19 dynamics in the UK to consider two competing SARS-CoV-2 lineages: VOC 202012/01 and preexisting variants. By fitting this model to a variety of data sources on infections, hospitalizations, and deaths across seven regions of England, we assessed different hypotheses for why the new variant appears to be spreading more quickly, estimated the severity of disease associated with the new variant, and evaluated control measures including vaccination and nonpharmaceutical interventions. Combining multiple lines of evidence allowed us to draw robust inferences.

**RESULTS:**

The rapid spread of VOC 202012/01 is not an artifact of geographical differences in contact behavior and does not substantially differ by age, sex, or socioeconomic stratum. We estimate that the new variant has a 43 to 90% higher reproduction number (range of 95% credible intervals, 38 to 130%) than preexisting variants. Similar increases are observed in Denmark, Switzerland, and the United States. The most parsimonious explanation for this increase in the reproduction number is that people infected with VOC 202012/01 are more infectious than people infected with a preexisting variant, although there is also reasonable support for a longer infectious period and multiple mechanisms may be operating. Our estimates of severity are uncertain and are consistent with anything from a moderate decrease to a moderate increase in severity (e.g., 32% lower to 20% higher odds of death given infection). Nonetheless, our mathematical model, fitted to data up to 24 December 2020, predicted a large surge in COVID-19 cases and deaths in 2021, which has been borne out so far by the observed burden in England up to the end of March 2021. In the absence of stringent nonpharmaceutical interventions and an accelerated vaccine rollout, COVID-19 deaths in the first 6 months of 2021 were projected to exceed those in 2020 in England.

**CONCLUSION:**

More than 98% of positive SARS-CoV-2 infections in England are now due to VOC 202012/01, and the spread of this new variant has led to a surge in COVID-19 cases and deaths. Other countries should prepare for potentially similar outcomes.

## Teaser

The major coronavirus variant that emerged at the end of 2020 in the UK is more transmissible than its predecessors and could spark resurgences.

## Graphical abstract

**Figure:**
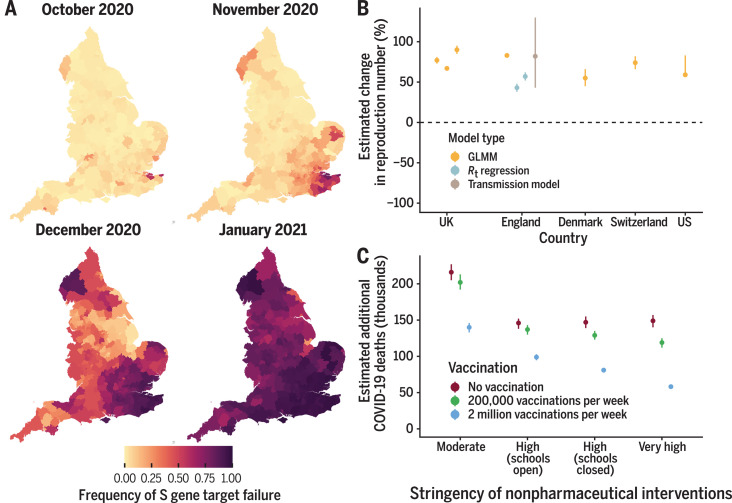
Impact of SARS-CoV-2 Variant of Concern 202012/01. (**A**) Spread of VOC 202012/01 (lineage B.1.1.7) in England. (**B**) The estimated relative transmissibility of VOC 202012/01 (mean and 95% confidence interval) is similar across the United Kingdom as a whole, England, Denmark, Switzerland, and the United States. (**C**) Projected COVID-19 deaths (median and 95% confidence interval) in England, 15 December 2020 to 30 June 2021. Vaccine rollout and control measures help to mitigate the burden of VOC 202012/01.

In December 2020, evidence began to emerge that a severe acute respiratory syndrome coronavirus 2 (SARS-CoV-2) variant, Variant of Concern 202012/01 (lineage B.1.1.7, henceforth VOC 202012/01), was rapidly outcompeting preexisting variants in southeast England (*1*). The variant increased in incidence during the second national lockdown in November 2020, which was mandated in response to a previous and unrelated surge in COVID-19 cases, and continued to spread after the lockdown despite ongoing restrictions in many of the most affected areas. Concern over this variant led the UK government to enact stronger restrictions in these regions on 20 December 2020 and eventually to impose a third national lockdown on 5 January 2021. As of 29 March 2021, VOC 202012/01 comprises roughly 95% of new SARS-CoV-2 infections in England and has now been identified in at least 114 countries (*2*). Our current understanding of effective pharmaceutical and nonpharmaceutical control of SARS-CoV-2 does not reflect the epidemiological and clinical characteristics of VOC 202012/01. Estimates of the growth rate, disease severity, and impact of this novel variant are crucial for informing rapid policy responses to this potential threat.

## Characteristics of the new variant

VOC 202012/01 is defined by 17 mutations (14 nonsynonymous point mutations and three deletions), of which eight are in the spike protein, which mediates SARS-CoV-2 attachment and entry into human cells. At least three mutations potentially affect viral function. Mutation N501Y is a key contact residue in the receptor binding domain and enhances virus binding affinity to human angiotensin-converting enzyme 2 (ACE2) (*3*, *4*). Mutation P681H is immediately adjacent to the furin cleavage site in spike, a known region of importance for infection and transmission (*5*, *6*). Deletion ∆H69/∆V70 in spike has arisen in multiple independent lineages of SARS-CoV-2, is linked to immune escape in immunocompromised patients, and enhances viral infectivity in vitro (*7*, *8*). This deletion is also responsible for certain commercial testing kits failing to detect the spike glycoprotein gene, and genomic data confirm that these S gene target failures in England are now overwhelmingly attributable to the new variant (*1*).

**Fig. 1:**
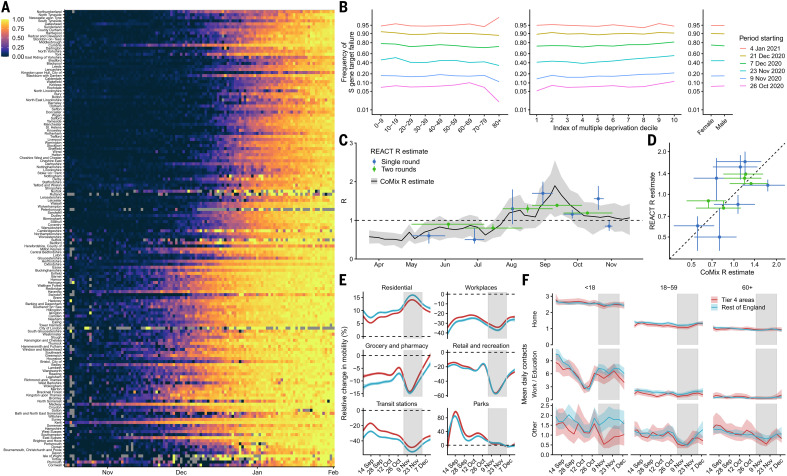
Rapid spread of VOC 202012/01 in England. (**A**) Proportion of S gene target failure among positive Pillar 2 community SARS-CoV-2 tests in upper-tier local authorities of England from 1 October 2020 to 10 January 2021, sorted by latitude. (**B**) Spread of S gene target failure by age, index of multiple deprivation decile (1 = most deprived), and sex within Greater London. (**C** and** D**) Estimates of *R*_0_ from CoMix social contact survey (*12*) compared to *R*_t_ estimates from REACT-1 prevalence survey (*9*) for England, with 90% CIs. *R*_t_ estimates based on single and aggregated REACT-1 survey rounds are shown. Horizontal error bars in (C) show the date range over which *R*_t_ was measured. (**E** and **F**) Percentage change (95% CI) in Google Mobility indices relative to baseline over time (E) and setting-specific mean contacts (95% CI) from the CoMix study (*12*) over time and by age for Tier 4 local authorities compared to the rest of England (F). Tier 4 local authorities are areas within the South East, East of England, and London regions that were placed under stringent restrictions from 20 December 2020 because of high prevalence of VOC 202012/01 and growing case rates. Gray shaded areas show the second national lockdown in England.

The proportion of COVID-19 cases attributable to VOC 202012/01 increased rapidly in all regions of England, following an initial expansion in the southeast (Fig. 1A), and spread at comparable rates among males and females and across age and socioeconomic strata (Fig. 1B). One potential explanation for the spread of VOC 202012/01 within England is a founder effect; that is, if certain regions had higher levels of transmission as a result of more social interactions, variants that were more prevalent within these regions could become more common overall. Changes in social contact patterns correlate closely with changes in transmission (*9*) (Fig. 1, C and D) and with COVID-19 burden in England (*10*). However, we did not find substantial differences in social interactions between regions of high and low VOC 202012/01 prevalence, as measured by Google mobility (*11*) and social contact survey data (*12*) from September to December 2020 (Fig. 1, E and F). Therefore, the apparent decoupling between contact rates and transmission in late 2020 may suggest altered transmission characteristics for VOC 202012/01.

## Measuring the new variant’s growth rate

**Fig. 2:**
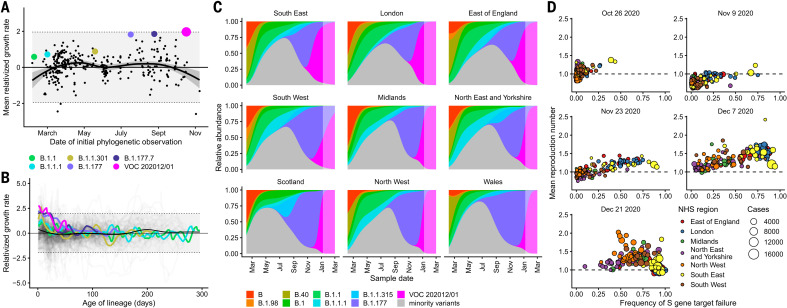
Measuring the growth rate of VOC 202012/01. (**A**) Average relativized growth rate (i.e., a measure of variant fitness relative to other variants present during the 31 days after initial phylogenetic observation of a given variant) for all lineages in the COG-UK dataset, highlighting many lineages that have risen to prominence including B.1.177, the lineage with the highest relative abundance during the initial phylogenetic observation of VOC 202012/01. The shaded regions show conservative 95% rejection intervals; VOC 202012/01 is the first strain to exceed this threshold of faster relativized growth. Although many lineages exhibit above-average rates of growth, VOC 202012/01 has had the highest average relativized growth of any lineage in the history of COG-UK surveillance of SARS-COV-2. (**B**) Plotting all lineages’ relativized growth rates [ρ(*t*)] as a function of lineage age with conservative 95% rejection intervals highlights the significantly faster growth of VOC 202012/01 relative to other lineages at comparable times since their initial observation. Later declines in VOC and B.1.177 correspond to highly uncertain estimates of growth rates for data that are yet to be backfilled, so these declines in ρ(*t*) are sensitive to the processing of future sequences from recent dates (fig. S1). (**C**) Muller plots of the relative abundances of the major SARS-CoV-2 variants in the UK, based on a multinomial spline fit to COG-UK sequence data (Table 1 and table S1, separate-slopes multinomial spline model). A model extrapolation until 1 March 2021 is shown (shaded area). Minority variants are 440 circulating SARS-CoV-2 variants of low abundance. Specific colors represent the same lineages in (A) to (C). (**D**) Mean reproduction number over 7-day periods in 149 upper-tier local authorities of England (colored by the NHS England region they are within) plotted against the weekly proportion of Pillar 2 community SARS-CoV-2 tests with S gene target failure shows the spread of VOC 202012/01, a corresponding increase in the reproduction number in each local authority, and the eventual impact of targeted government restrictions from 20 December 2020. Testing data are shown for the week after the reproduction number estimates to account for delays from infection to test.

VOC 202012/01 appears unmatched in its ability to outcompete other SARS-CoV-2 lineages in England. Analyzing the COG-UK dataset (*13*), which comprises more than 150,000 sequenced SARS-CoV-2 samples from across the UK, we found that the relative population growth rate of VOC 202012/01 in the first 31 days after its initial phylogenetic observation was higher than that of all 307 other lineages with enough observations to obtain reliable growth-rate estimates (Fig. 2A and fig. S1). Although the relative growth rate of VOC 202012/01 has declined slightly over time, it remains among the highest of any lineage as a function of lineage age (Fig. 2B), and the lineage continues to expand.

To quantify the growth advantage of VOC 202012/01, we performed a series of multinomial and logistic regression analyses on COG-UK data. A time-varying multinomial spline model estimates an increased growth rate for VOC 202012/01 of +0.104 day^–1^ [95% confidence interval (CI), 0.100 to 0.108] relative to the previously dominant lineage, B.1.177 (Table 1, model 1a; Fig. 2C; and figs. S2 and S3). Assuming a generation interval of 5.5 days (*14*), this corresponds to a 77% (95% CI, 73 to 81%) increase in the reproduction number *R*. The growth advantage of VOC 202012/01 persists under more conservative model assumptions (Table 1, model 1b; fig. S4), is consistent across all regions of the UK (table S1, model 2a; fig. S5), and is similar when measured from S gene target failures among community COVID-19 tests instead of COG-UK sequence data (Table 1, model 2h; fig. S6). Data from other countries yield similar results: We estimate that *R* for VOC 202012/01 relative to other lineages is 55% (95% CI, 45 to 66%) higher in Denmark, 74% (95% CI, 66 to 82%) higher in Switzerland, and 59% (95% CI, 56 to 63%) higher in the United States, with consistent rates of displacement across regions within each country (Table 1, models 3a to 3c; figs. S6 and S7).

**Table 1:** Estimates of increased reproduction number for VOC 202012/01. Means and 95% CIs (GLMM) or 95% CrIs (*R*_t_ regression, transmission model) are shown. GLMM models do not estimate a baseline growth rate or reproduction number. Increases in the reproduction number assume a generation interval of 5.5 days. See table S1 for full details.

**Model type**	**Model **	**Model assumptions**	**Data**	**Geography**	**Baseline ** **growth rate**	**Additive ** **increase in ** **growth rate, ∆*r***	**Baseline ** **reproduction ** **number**	**Multiplicative ** **increase in ** **reproduction ** **number**
GLMM	1a	Separate-slopes multinomial spline model*	Sequence	Regions of UK	—	0.104 [0.100, 0.108]	—	77% [73, 81]
GLMM	1b	Common-slope multinomial model*	Sequence	Lower-tier local authorities of UK	—	0.093 [0.091, 0.095]	—	67% [65, 69]
GLMM	2h	Separate-slope binomial spline model†	S gene target failure‡	Regions of England	—	0.109 [0.107, 0.111]	—	83% [81, 84]
*R*_t_ regression	4a	Regional time- varying baseline	S gene target failure	Upper-tier local authorities of England	0.007 [0.002, 0.012]	0.067 [0.060, 0.073]	1.04 [1.01, 1.07]	43% [38, 48]
*R*_t_ regression	4b	Regional static baseline	S gene target failure	Upper-tier local authorities of England	0.007 [0.002, 0.012]	0.085 [0.079, 0.091]	1.04 [1.01, 1.07]	57% [52, 62]
Transmission model	5a	Increased transmissibility	S gene target failure‡	Regions of England	–0.001 [–0.017, 0.012]	0.118 [0.067, 0.168]	1.01 [0.94, 1.09]	82% [43, 130]
GLMM	3a	Common-slope binomial model†	Sequence	Regions of Denmark	—	0.080 [0.067, 0.092]	—	55% [45, 66]
GLMM	3b	Common-slope binomial model†	Sequence + RT-PCR rescreening	Regions of Switzerland	—	0.101 [0.092, 0.109]	—	74% [66, 82]
GLMM	3c	Common-slope binomial model†	S gene target failure‡	States of USA	—	0.084 [0.080, 0.088]	—	59% [56, 83]

*VOC 202012/01 versus B.1.177.

†VOC 202012/01 versus all other variants.

‡Binomial counts adjusted for the true positive rate (proportion of S gene target failures that are VOC 202012/01), estimated from misclassification model (for UK) or a binomial GLMM fitted to sequencing data of S gene target failures (for US).

As an alternative approach, we performed a regression analysis of reproduction numbers estimated from case data against the frequency of S gene target failure in English upper-tier local authorities (Fig. 2D), using local control policies and mobility data as covariates and including a time-varying spline to capture any unmeasured confounders. This yielded an estimated increase in *R* for VOC 202012/01 of 43% (95% CI, 38 to 48%), increasing to a 57% (95% CI, 52 to 62%) increase if the spline was not included (Table 1, models 4a and 4b). The various statistical models we fitted yield slightly different estimates for the growth rate of VOC 202012/01, reflecting different assumptions and model structures, but all identify a substantially increased growth rate (table S1).

## Mechanistic hypotheses for the rapid spread

To understand possible biological mechanisms for the faster spread of VOC 202012/01 relative to preexisting variants, we extended an age-structured and regionally structured mathematical model of SARS-CoV-2 transmission (*10*, *15*) to consider two co-circulating variants (fig. S8 and tables S2 and S3). The model uses Google mobility data (*11*), validated by social contact surveys (*10*), to capture changes in contact patterns over time for each region of England. We created five versions of the model, each including one alternative parameter capturing a potential mechanism.

The hypotheses we tested are as follows. First, observations of lower cycle threshold (Ct) values (*16*–*18*)—that is, higher viral load—support the idea that VOC may be more transmissible per contact with an infectious person than preexisting variants (hypothesis 1). Second, longitudinal testing data (*17*) suggest that VOC may be associated with a longer period of viral shedding and hence a potentially longer infectious period (hypothesis 2). Third, the ∆H69/∆V70 deletion in spike contributed to immune escape in an immunocompromised patient (*7*), which suggests that immunity to preexisting variants may afford reduced protection against infection with VOC (hypothesis 3). Fourth, the initial spread of VOC during the November 2020 lockdown in England, during which schools were open, suggests that children may be more susceptible to infection with VOC than with preexisting variants (hypothesis 4). Children are typically less susceptible to SARS-CoV-2 infection than adults (*19*, *20*), possibly because of immune cross-protection due to other human coronaviruses (*21*), which could be less protective against VOC. Finally, VOC could have a shorter generation time than preexisting variants (hypothesis 5). A shorter generation time could account for an increased growth rate without requiring a higher reproduction number, which would make control of VOC 202012/01 through social distancing measures relatively easier to achieve.

**Fig. 3:**
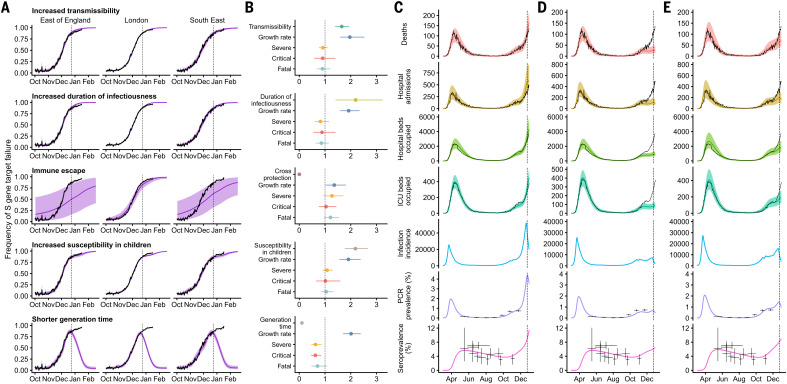
Comparison of possible biological mechanisms underlying the rapid spread of VOC 202012/01. Each row shows a different assumed mechanism. (**A**) Relative frequency of VOC 202012/01 (black line and ribbon respectively denote observed S gene target failure frequency with 95% binomial credible interval; purple line and ribbon respectively denote mean and 95% credible interval from model fit). (**B**) Posterior estimates (mean and 95% credible intervals) for relative odds of hospitalization (severe illness), relative odds of ICU admission (critical illness), relative odds of death (fatal illness), growth rate as a multiplicative factor per week [i.e., exp(7·∆*r*)], and the parameter that defines the hypothesized mechanism; all parameters are relative to those estimated for preexisting variants. (**C** to **E**) Illustrative model fits for the South East NHS England region: (C) fitted two-strain increased transmissibility model with VOC 202012/01 included; (D) fitted two-strain increased transmissibility model with VOC 202012/01 removed; (E) fitted single-strain model without emergence of VOC 202012/01. Black lines denote observed data; error bars denote the date range and 95% credible intervals for observed PCR prevalence and seroprevalence; colored lines and ribbons denote median and 95% credible intervals from model fit.

We fit each model to time series of COVID-19 deaths, hospital admissions, hospital and ICU bed occupancy, polymerase chain reaction (PCR) prevalence, seroprevalence, and the proportion of community SARS-CoV-2 tests with S gene target failure across the three most heavily affected NHS England regions, over the period 1 March to 24 December 2020 (Fig. 3 and figs. S9 to S14). We assessed models using deviance information criteria (DIC) and compared model predictions to observed data for the 14 days after the fitting period (i.e., 25 December 2020–7 January 2021). Of the five hypotheses assessed, hypothesis 1 (increased transmissibility) had the lowest (i.e., best) combined DIC and predictive deviance. Hypothesis 2 (longer infectious period) and hypothesis 4 (increased susceptibility in children) also fitted the data well, although hypothesis 4 is not well supported by household secondary attack rate data (fig. S15) or by age-specific patterns of S gene target failure in the community (fig. S16), neither of which identify a substantial increase in susceptibility among children. Hypothesis 3 (immune escape) and hypothesis 5 (shorter generation time) fit poorly (Fig. 3A and table S4). In particular, hypothesis 5 predicted that the relative frequency of VOC 202012/01 should have dropped during stringent restrictions in late December 2020, because when two variants have the same effective reproduction number *R*_t_ < 1 but different generation times, infections decline faster for the variant with the shorter generation time.

We fitted a combined model incorporating the five hypotheses above, but it was not able to identify a single consistent mechanism across NHS England regions; hence, a wide range of parameter values are compatible with the observed growth rate of VOC 202012/01 (fig. S14). On the basis of our analysis, we identify increased transmissibility as the most parsimonious model, but we emphasize that the five mechanisms explored here are not mutually exclusive and may be operating in concert.

The increased transmissibility model does not identify a clear increase or decrease in the severity of disease associated with VOC 202012/01, finding similar odds of hospitalization given infection [odds ratio, 0.92; 95% credible interval (CrI), 0.77 to 1.10], critical illness [odds ratio, 0.90 (CrI, 0.58 to 1.40)], and death [odds ratio, 0.90 (0.68 to 1.20)] when the model was fitted to the three most heavily affected NHS England regions (Fig. 3B). These estimates should be treated with caution, as we would not expect to identify a clear signal of severity when fitting to data up to 24 December 2020, given delays between infection and hospitalization or death. However, the fitted model finds strong evidence of higher relative transmissibility, estimated at 65% (CrI, 39 to 93%) higher than preexisting variants for the three most heavily affected NHS England regions, or 82% (CrI, 43 to 130%) when estimated across all seven NHS England regions (Table 1, model 5a). These estimates of increased transmissibility are consistent with our statistical estimates and with a previous estimate of a 70% increased reproduction number for VOC 202012/01 (*16*). This model reproduces observed epidemiological dynamics for VOC 202012/01 (Fig. 3C and fig. S17). Without the introduction of a new variant with a higher growth rate, the model is unable to reproduce observed dynamics (Fig. 3, D and E, and figs. S17 to S19); these findings lend further support to the idea that changing contact patterns do not explain the spread of VOC 202012/01.

## Implications for COVID-19 dynamics in England

Using the best-performing transmission model (increased transmissibility) fitted to all seven NHS England regions, we compared projected epidemic dynamics under different assumptions about control measures from mid-December 2020 to the end of June 2021. We compared four scenarios for nonpharmaceutical interventions (NPIs) introduced on 1 January 2021: (i) a moderate-stringency scenario with mobility levels as observed in the first half of October 2020; (ii) a high-stringency scenario with mobility levels as observed during the second national lockdown in England in November 2020, with schools open; (iii) the same high-stringency scenario, but with schools closed until 15 February 2021; and (iv) a very-high-stringency scenario with mobility levels as observed during the first national lockdown in early April 2020, with schools closed (fig. S20). In combination with these NPI scenarios, we considered three vaccination scenarios: no vaccinations; 200,000 vaccinations per week; and 2 million vaccinations per week. We assumed that vaccine rollout starts on 1 January 2021 and that vaccinated individuals have a 95% lower probability of disease and a 60% lower probability of infection than unvaccinated individuals. For simplicity, we assume that vaccine protection is conferred immediately upon receipt of one vaccine dose. Note that these projections serve as indicative scenarios rather than formal predictive forecasts.

**Fig. 4:**
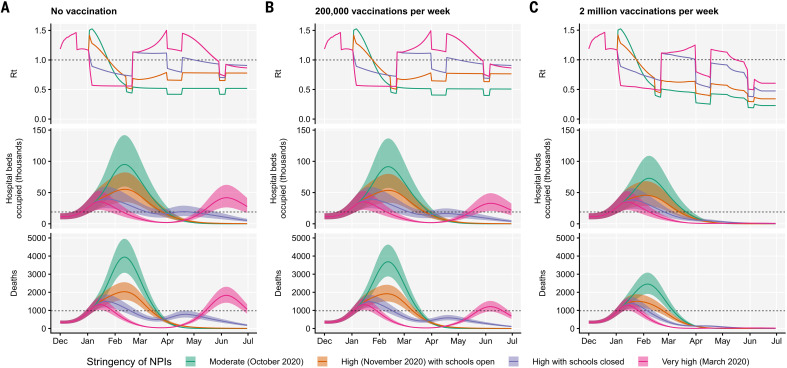
Projections of epidemic dynamics under different control measures. We compare four alternative scenarios for nonpharmaceutical interventions from 1 January 2021: (i) mobility returning to levels observed during relatively moderate restrictions in early October 2020; (ii) mobility as observed during the second lockdown in England in November 2020, then gradually returning to October 2020 levels from 1 March to 1 April 2021, with schools open; (iii) as (ii), but with schools closed until 15 February 2021; (iv) as (iii), but with a lockdown of greater stringency as observed in March 2020 (fig. S20). (**A**) Without vaccination. (**B**) With 200,000 people vaccinated per week. (**C**) With 2 million people vaccinated per week. We assume that vaccination confers 95% vaccine efficacy against disease and 60% vaccine efficacy against infection, and that vaccination starts on 1 January 2021 with vaccine protection starting immediately upon receipt. This is intended to approximate the fact that vaccination started in early December, with full protection occurring after a time lag and potentially after a second dose. Vaccines are given first to people aged 70+ until 85% coverage is reached in this age group, then to people aged 60+ until 85% coverage is reached in this age group, continuing into younger age groups in 10-year decrements. Resurgences starting in March 2021 are due to the relaxation of nonpharmaceutical interventions, including reopening schools (fig. S20). Median and 95% credible intervals are shown. The dashed lines in rows 2 and 3 show peak hospitalizations and deaths from the first COVID-19 wave in England (April 2020).

Regardless of control measures, all regions of England were projected to experience a new wave of COVID-19 cases and deaths in early 2021, peaking in February 2021 if no substantial control measures were introduced, or in mid-January 2021 if strong control measures succeeded in reducing *R* below 1 (Fig. 4A). In the absence of substantial vaccine rollout, the numbers of COVID-19 cases, hospitalizations, ICU admissions, and deaths in the first 6 months of 2021 were projected to exceed those in 2020, even with stringent NPIs in place (Table 2). Implementing more stringent measures in January 2021 (scenarios iii and iv) led to a larger rebound in cases when simulated restrictions were lifted in March 2021, particularly in those regions that had been least affected up to December 2020 (fig. S21). However, more stringent measures may buy time to reach more widespread population immunity through vaccination. Vaccine rollout further mitigated transmission, although the impact of vaccinating 200,000 people per week—similar in magnitude to the rates reached in December 2020—was relatively small (Fig. 4B and fig. S22). An accelerated uptake of 2 million people fully vaccinated per week (i.e., 4 million doses for a two-dose vaccine) had a much more substantial impact (Fig. 4C and fig. S23). However, accelerated vaccine rollout had a relatively limited impact on peak burden, as the peak was largely mediated by the stringency of NPIs enacted in January 2021, before vaccination had much of an impact. The primary benefit of accelerated vaccine rollout lies in helping to avert a resurgence of cases after the relaxation of NPIs, and in reducing transmission after the peak burden has already been reached.

**Table 2:** Summary of projections for England, 15 December 2020 to 30 June 2021. Median and 95% credible intervals are indicated.

	**Moderate** (**October 2020**)	**High** (**November 2020**) **with schools open**	**High with schools closed**	**Very high** (**March 2020**)
***No vaccination***				
Peak ICU (relative to 1st wave)	274% (256–292%)	162% (151–172%)	130% (122–136%)	119% (112–124%)
Peak ICU bed requirement	9,980 (9,330–10,600)	5,880 (5,490–6,280)	4,720 (4,450–4,960)	4,310 (4,070–4,530)
Peak deaths	3,960 (3,730–4,200)	2,050 (1,920–2,160)	1,500 (1,440–1,570)	1,830 (1,670–2,000)
Total admissions	635,000 (604,000–659,000)	454,000 (432,000–472,000)	448,000 (425,000–466,000)	450,000 (425,000–472,000)
Total deaths	216,000 (205,000–227,000)	146,000 (138,000–152,000)	147,000 (139,000–155,000)	149,000 (140,000–157,000)
***200,000 vaccinations per week***				
Peak ICU (relative to 1st wave)	269% (252–287%)	160% (149–170%)	130% (122–136%)	118% (112–124%)
Peak ICU bed requirement	9,790 (9,150–10,400)	5,810 (5,430–6,200)	4,710 (4,450–4,950)	4,310 (4,070–4,520)
Peak deaths	3,700 (3,500–3,920)	1,930 (1,820–2,040)	1,490 (1,430–1,550)	1,320 (1,280–1,380)
Total admissions	610,000 (580,000–634,000)	438,000 (416,000–454,000)	415,000 (394,000–430,000)	394,000 (373,000–413,000)
Total deaths	202,000 (192,000–213,000)	137,000 (130,000–143,000)	129,000 (123,000–135,000)	119,000 (112,000–125,000)
***2 million vaccinations per week***				
Peak ICU (relative to 1st wave)	236% (221–252%)	149% (139–158%)	128% (121–134%)	118% (111–124%)
Peak ICU bed requirement	8,590 (8,050–9,170)	5,400 (5,070–5,760)	4,650 (4,390–4,880)	4,290 (4,060–4,500)
Peak deaths	2,470 (2,330–2,610)	1,510 (1,450–1,580)	1,390 (1,340–1,450)	1,290 (1,250–1,340)
Total admissions	483,000 (459,000–502,000)	353,000 (337,000–366,000)	277,000 (265,000–287,000)	190,000 (182,000–197,000)
Total deaths	140,000 (133,000–146,000)	98,900 (94,600–103,000)	81,000 (77,600–84,200)	58,200 (56,100–60,300)

As a sensitivity analysis, we also ran model projections with a seasonal component such that transmission is 20% higher in winter than in summer (*22*), but this did not qualitatively affect our results (fig. S24 and table S5).

## Discussion

Combining multiple behavioral and epidemiological data sources with statistical and dynamic modeling, we estimated that the SARS-CoV-2 variant VOC 202012/01 has a 43 to 90% (range of 95% CrIs, 38 to 130%) higher reproduction number than preexisting variants of SARS-CoV-2 in England, assuming no changes to the generation interval. On the basis of early population-level data, we were unable to identify whether the new variant is associated with higher disease severity. Theoretical considerations suggest that, in some cases, natural selection may favor reduced severity of disease in pathogens (*23*). For this to be true, however, the pathogen’s infectious period must be truncated by disabling symptoms or death often enough that a less-virulent mutant generates more secondary infections despite potentially being less transmissible per contact, to the extent that decreased virulence and decreased transmissibility are consequences of the same mutation (e.g., one that decreases viral load). It is far from clear that this condition holds for SARS-CoV-2, given substantial transmission before the onset of severe symptoms. Regardless, without strengthened controls, there is a clear risk that future epidemic waves may be larger—and hence associated with greater burden—than previous waves. The UK government initiated a third national lockdown on 5 January 2021 in response to the rapid spread of VOC 202012/01, including school closures. Educational settings are among the largest institutions linked to SARS-CoV-2 clusters that remained open during the November 2020 lockdown (*24*), which means that the enacted school and university closures may have substantially assisted in reducing the burden of COVID-19 in early 2021.

The increase in transmission associated with VOC 202012/01 has crucial implications for vaccination. First, it means that prompt and efficient vaccine delivery and distribution are even more important to reduce the impact of the pandemic in the near future. Increased transmission resulting from VOC 202012/01 will raise the herd immunity threshold, so that the potential burden of SARS-CoV-2 is larger and higher vaccine coverage will be required to achieve herd immunity. It is therefore extremely concerning that VOC 202012/01 has spread to at least 114 countries globally (*2*). Although VOC 202012/01 was first identified in England, a rapidly spreading variant has also been detected in South Africa (*25*, *26*), where there was a marked increase in transmission in late 2020. Another variant exhibiting immune escape has emerged in Brazil (*27*, *28*). Thus, vaccination timelines will also be a crucial determinant of future burden in other countries where similar new variants are present. Second, there is a need to assess how VOC 202012/01 and other emerging lineages affect the efficacy of vaccines (*29*, *30*). Vaccine developers may need to consider developing formulations with variant sequences, and they may want to initiate post-licensure studies to detect differences in efficacy between the preexisting and new variants. Licensing authorities may need to clarify abbreviated pathways to marketing for vaccines that involve altering strain formulation without any other changes to their composition.

There are limitations to our analysis. We have considered a small number of intervention and vaccination scenarios, which should not be regarded as the only available options for policy-makers. Our transmission model does not explicitly capture nursing home or hospital transmission of SARS-CoV-2, and we fit the model to each region of England separately rather than pooling information across regions and explicitly modeling transmission between regions. There are also uncertainties in the choice of model used to generate these predictions, and the exact choice will yield differences in the measures needed to control the epidemic. We note that even without increased susceptibility of children to VOC 202012/01, the more efficient spread of the variant implies that the difficult societal decision of closing schools will be a key public health question for multiple countries in the months ahead.

We only assess relative support in the data for the mechanistic hypotheses proposed, but there may be other plausible mechanisms driving the resurgence of cases that we did not consider, and we have not identified the specific combination of mechanisms driving the increased transmission of VOC 202012/01. We identify increased transmissibility as the most parsimonious mechanistic explanation for the higher growth rate of VOC 202012/01, but a longer infectious period also fits the data well (table S4) and is supported by longitudinal testing data (*17*). Our conclusions about school closures were based on the assumption that children had reduced susceptibility and infectiousness relative to adults (*19*), but the precise values of these parameters and the impact of school closures remain the subject of scientific debate (*31*). We based our assumptions about the efficacy of NPIs on the measured impact on mobility of previous national lockdowns in England, but the impact of policy options cannot be predicted with certainty.

Despite these limitations, we found strong evidence that VOC 202012/01 is spreading substantially faster than preexisting SARS-CoV-2 variants. Our modeling analysis suggests that this difference could be explained by an overall higher infectiousness of VOC 202012/01, but not by a shorter generation time or immune escape alone. Further experimental work will provide insight into the biological mechanisms for our observations, but given our projections of a rapid rise in incidence from VOC 202012/01—and the detection of other novel and highly transmissible variants (*25*–*28*)—there is an urgent need to consider what new approaches may be required to sufficiently reduce the ongoing transmission of SARS-CoV-2.

## Materials and methods

### Summary of control measures in England in late 2020

After a resurgence of cases in September and October 2020, a second national lockdown was implemented in England, from 5 November to 2 December 2020. Restrictions included a stay-at-home order with exemptions for exercise, essential shopping, obtaining or providing medical care, education, and work for those unable to work from home. Schools were kept open. Non-essential shops and retail and leisure venues were required to close. Pubs, bars, and restaurants were allowed to offer takeaway services only. After the second national lockdown, regions in England were assigned to tiered local restrictions according to medium, high, and very high alert levels (Tiers 1, 2, and 3). In response to rising cases in southeast England and concerns over VOC 202012/01, the UK government announced on 19 December 2020 that a number of regions in southeast England would be placed into a new, more stringent “Tier 4,” corresponding to a Stay at Home alert level. Tier 4 restrictions were broadly similar to the second national lockdown restrictions. As cases continued to rise and VOC 202012/01 spread throughout England, on 5 January 2021 a third national lockdown was introduced in England, with schools and universities closed and individuals advised to stay at home, with measures to be kept in place until at least mid-February 2021.

### Data sources

To assess the spread of VOC 202012/01 in the UK, we used publicly available sequencing-based data from the COG-UK Consortium (*13*) (5 February 2020–6 January 2021) and Pillar 2 SARS-CoV-2 testing data provided by Public Health England (1 October 2020–7 January 2021) for estimating the frequency of S gene target failure in England. COG-UK sequencing data for Northern Ireland were excluded because of low sample sizes.

To assess the spread of VOC 202012/01 in Denmark, Switzerland, and the US, we used publicly available sequence data giving the incidence of VOC 202012/01 aggregated by week and region provided by the Danish Covid-19 Genome Consortium and the Statens Serum Institut (*32*) (15 October 2020–28 January 2021), sequence and RT-PCR 501Y.V1 rescreening data giving the incidence of VOC 202012/01 in different regions of Switzerland provided by Christian Althaus and Tanja Stadler and the Geneva University Hospitals, the Swiss Viollier Sequencing Consortium from ETH Zürich, the Risch laboratory, the University Hospital Basel, the Institute for Infectious Diseases, University of Bern, and the Swiss National Covid-19 Science Task Force (*33*, *34*) (2 November 2020–11 February 2021), and publicly available US nationwide Helix SARS-CoV-2 Surveillance data, comprising both S gene target failure data and randomly selected S-negative samples that were sequenced to infer the proportion of S-negative samples that were the VOC (*35*, *36*) (6 September–11 February 2020).

To estimate mobility, we used anonymized mobility data collected from smartphone users by Google Community Mobility (*11*). Percentage change in mobility per day was calculated for each lower-tier local authority in England, and a generalized additive model with a spline for time was fitted to these observations to provide a smoothed effect of the change in mobility over time (Fig. 1C).

To estimate social contact rates (Fig. 1D), we used data on reported social contacts from the CoMix survey (*12*), which is a weekly survey of face-to-face contact patterns, taken from a sample of ~2500 individuals broadly representative of the UK population with respect to age and geographical location. We calculated the distribution of contacts using 1000 bootstrap samples with replacement from the raw data. Bootstrap samples were calculated at the participant level, then all observations for those participants were included in a sample to respect the correlation structure of the data. We collected data in two panels that alternated weekly; therefore, we calculated the mean smoothed over the 2-week intervals to give a larger number of participants per estimate and to account for panel effects. We calculated the mean number of contacts (face-to-face conversational contact or physical contact) in the settings “home,” “work,” “education” (including childcare, nurseries, schools, and universities and colleges), and “other” settings. We calculated the mean contacts by age group and area of residence (those areas that were subsequently placed under Tier 4 restrictions on 20 December 2020 as they were experiencing high and rapidly increasing incidence, and those areas of England that were not placed under these restrictions). The mean number of contacts was influenced by a few individuals who reported very high numbers of contacts (often in a work context). The means shown here were calculated by truncating the maximum number of contacts recorded at 200 per individual per day. We compared *R*_t_ estimates derived from CoMix (*12*) to those derived from the REACT-1 prevalence survey (*9*) for England.

### Statistical methods in brief

*Growth of VOC 202012/01 after initial phylogenetic observation*. For each lineage *i* in the COG-UK dataset, we pooled the number of sequences observed within that lineage across the UK for every day, *t*, yielding integer-valued sequence counts *N*(*i*, *t*). We estimated the time-varying exponential growth rates of cases of each strain, *r*(*i*, *t*), using a negative binomial state-space model correcting for day-of-week effects whose dispersion parameter was optimized for each strain by marginal likelihood maximization. We defined the relativized growth rate of a lineage *i* at time *t* as ρ(*i*, *t*) = $\left[r\left(i,t\right)-\bar{r}\left(t\right)\right]/\sigma_{r}\left(t\right)$, where $\bar{r}\left(t\right)$ is the average growth rate of all circulating strains at time *t* and σ*_r_*(*t*) is the standard deviation of growth rates across all lineages at time *t*, such that ρ(*i*, *t*) is analogous to a z-statistic or Wald-type statistic and allows comparison of growth rate differences across time when the average growth rate and scale of growth rate differences varies.

*Competitive advantage and increased growth rate of VOC-202012/01.* To estimate the increase in growth rate of VOC 202012/01, we fitted a set of multinomial and binomial generalized linear mixed models (GLMMs), in which we estimated the rate by which the VOC displaces other resident SARS-CoV-2 variants across different regions in the UK, based on both the COG-UK sequence data and the S gene target failure data. In the analysis of the S gene target failure data, binomial counts were adjusted for the true positive rate. For comparison, we also calculated the growth advantage of the VOC in Denmark, Switzerland, and the US based on both sequencing and S gene target failure data. All models took into account sample date and region, plus (if desired) their interaction, and all mixed models took into account possible overdispersion and for the UK further included local-tier local authority as a random intercept. From these models, we estimated the difference in Malthusian growth rate between other competing variants Δ*r*, as well as the expected multiplicative increase in basic reproduction number *R*_t_ and infectiousness, assuming unaltered generation time, which can be shown to be equal to exp(∆*r.T*), where *T* is the mean generation interval. The multiplicative increase being equal to exp(∆*r.T*) is an approximation that holds for a delta-distributed generation interval, but we show in the supplementary materials that this is a good approximation for the gamma-distributed generation interval that we assume. In our calculations, we used estimated SARS-CoV-2 mean generation times *T* of either 5.5 days (*14*) (Table 1) or 3.6 days (*37*, *38*) (table S1).

*R*_t_* analysis.* We calculated the weekly proportion of positive tests that were S gene–negative out of all positive tests that tested for the S gene by English upper-tier local authority. We used reproduction number estimates obtained using the method described in (*37*) and (*39*) and implemented in the EpiNow2 R package (*40*), downloaded from https://github.com/epiforecasts/covid-rt-estimates/blob/master/subnational/united-kingdom-local/cases/summary/rt.csv. We then built a separate model of the expected reproduction number in UTLA *i* during week *t* starting in the week beginning 5 October 2020 as a function of local restrictions, mobility indicators, residual temporal variation, and proportion of positive tests with S gene target failure. The residual temporal variation is modeled either as a region-specific thin-plate regression spline (“Regional time-varying”) or a static regional parameter (“Regional static”). The key estimand is the relative change in reproduction number in the presence of S gene target failure that is not explained by any of the other variables.

### Transmission dynamic model

We extended a previously developed modeling framework structured by age (in 5-year age bands, with no births, deaths, or aging due to the short time scales modeled) and by geographical region (*10*, *15*) to include two variants of SARS-CoV-2 (VOC 202012/01 and non-VOC 202012/01). The model is a discrete-time deterministic compartmental model that allows for arbitrary delay distributions for transitions between compartments. We fitted this model to multiple regionally stratified data sources across the seven NHS England regions as previously: deaths, hospital admissions, hospital bed occupancy, ICU bed occupancy, daily incidence of new infections, PCR prevalence of active infection, seroprevalence, and daily frequency of VOC 202012/01 across each of the regions as measured by S gene target failure frequency corrected for false positives. The model assumes that individuals with clinical symptoms are more infectious than individuals with subclinical infection (*19*). We assume that vaccinated individuals have a lower probability of both clinical and subclinical infection (fig. S9), but that vaccinated individuals who do develop clinical or subclinical infection are as infectious as unvaccinated individuals with clinical or subclinical infection. To model school closure, we removed all school contacts from our contact matrix based on POLYMOD data and varying over time according to Google Mobility indices, as described (*10*). See supplementary materials for details of Bayesian inference including likelihood functions and prior distributions.

Our individual transmission model fits to separate NHS regions of England produced independent estimates of parameters such as relative transmissibility and differences in odds of hospitalization or death resulting from infection with VOC 202012/01. To produce overall estimates for these parameters, we modeled posterior distributions from individual NHS regions as draws from a mixture distribution, comprising a normally distributed top-level distribution from which central estimates for each NHS region are drawn. We report the mean and credible intervals of the top-level distribution when reporting model posterior estimates for England.

In model fitting, we assume that our deterministic transmission model approximates the expectation over stochastic epidemic dynamics. This is not exact (*41*), but the error in this approximation is small for the population-level processes we are modeling, as it decays with 1/*N* (*42*). This approach is well developed for state-space models of communicable disease dynamics that fit an epidemic process to observed data via a stochastic observation process.

### Apparent growth of VOC 202012/01 not a result of testing artifacts

The apparent frequency of VOC 202012/01 could be inflated relative to reality if this variant leads to increased test-seeking behavior (e.g., if it leads to a higher rate of symptoms than preexisting variants). However, this would not explain the growth in the relative frequency of VOC 202012/01 over time. Mathematically, if variant 1 has growth rate *r*_1_ and variant 2 has growth rate *r*_2_, the relative frequency over time is *a*_2_ exp(*r*_2_*t*)/[*a*_1_ exp(*r*_1_*t*) + *a*_2_ exp(*r*_2_*t*)], where *a*_1_ and *a*_2_ are the frequency of variants 1 and 2, respectively, at time *t* = 0. However, if variant 1 has probability *x* of being reported and variant 2 has probability *y*, and both have growth rate *r*, the relative frequency over time is *a*_2_ *y* exp(*rt*)/[*a*_1_ *x* exp(*rt*) + *a*_2_ *y* exp(*rt*)], which is constant.

## Supplementary Materials

science.sciencemag.org/content/372/6538/eabg3055/suppl/DC1 Materials and Methods Supplementary Text Figs. S1 to S24 Tables S1 to S6 References (*44*–*75*) MDAR Reproducibility Checklist
